# Bacterial growth stage determines the yields, protein composition, and periodontal pathogenicity of *Porphyromonas gingivalis* outer membrane vesicles

**DOI:** 10.3389/fcimb.2023.1193198

**Published:** 2023-10-11

**Authors:** Hongchen Mao, Ting Gong, Yuting Sun, Shiyao Yang, Xin Qiao, Deqin Yang

**Affiliations:** ^1^ Department of Endodontics, Stomatological Hospital of Chongqing Medical University, Chongqing, China; ^2^ Stomatological Hospital of Chongqing Medical University, Chongqing Key Laboratory of Oral Diseases and Biomedical Sciences, Chongqing, China; ^3^ Chongqing Municipal Key Laboratory of Oral Biomedical Engineering of Higher Education, Chongqing, China; ^4^ Chongqing Key Laboratory of Oral Diseases and Biomedical Sciences, Chongqing, China

**Keywords:** *Porphyromonas gingivalis*, outer membrane vesicles, periodontal pathogenicity, rat periodontitis model, caspase-1/NLRP3/IL-1β

## Abstract

**Introduction:**

*P. gingivalis* (W83), as the keystone pathogen in chronic periodontitis, has been found to be tightly bound to systemic diseases. Outer membrane vesicles (OMVs) produced by *P. gingivalis* (W83) are thought to serve key functions in bacterial virulence and pathogenicity. This study aims to comprehend the biological functions of *P. gingivalis* OMVs isolated from different growth stages by comparing their physicochemical properties and pathogenicity.

**Methods:**

Protein composition was analyzed via isotope-labeled relative and absolute quantification (iTRAQ). Macrophage polarization and the expression of IL-6 and IL-1β were detected. The proliferation, migration, osteogenic differentiation, and IL-1b/NLRP3 expression of periodontal ligament stem cells (PDLSCs) were evaluated. *P. gingivalis*/*P. gingivalis* OMVs-induced periodontal models were also constructed in Sprague Dawley rats.

**Results:**

The protein composition of *P. gingivalis* OMVs isolated from different growth stages demonstrated obvious differences ranging from 25 KDa to 75 KDa. In the results of flow cytometry, we found that in vitro experiments the M1 subtype of macrophages was more abundant in the late-log OMVs and stationary OMVs groups which boosted the production of inflammatory cytokines more than pre-log OMVs. Compared to pre-log OMVs, late-log OMVs and stationary OMVs had more pronounced inhibitory effects on proliferation, migration, and early osteogenesis of PDLSCs. The NLRP3 inflammasome was activated to a larger extent in the stationary OMVs group. Micro-computed tomography (Micro CT), hematoxylin-eosin staining (HE), and tartrate acid phosphatase (TRAP) results showed that the periodontal damage in the stationary OMVs group was worse than that in the pre-log OMVs and late-log OMVs group, but almost equal to that in the positive control group (*P. gingivalis*).

**Discussion:**

In general, both in vivo and in vitro experiments showed that late-log OMVs and stationary OMVs have more significant pathogenicity in periodontal disease.

## 1 Introduction

Chronic periodontitis is a localized inflammatory disease caused by dysbiosis of the oral microbial community, with 11% of the world’s population suffering (Blasco-Baque et al., 2017; Pan et al., 2019; Bao et al., 2022). It mainly causes the chronic and progressive destruction of periodontal supporting tissue and eventually leads to the loosening and loss of teeth. Simultaneously, there is growing evidence of an inextricable link between periodontitis and systemic disorders, including but not limited to diabetes (Liccardo et al., 2019), rheumatoid arthritis (Priyamvara et al., 2020), adverse pregnancy outcomes (Bobetsis et al., 2020), Alzheimer’s disease (Kamer et al., 2020), and others. Among all bacteria within the oral cavity, *P. gingivalis*, a gram-negative bacterium, is widely regarded as the key pathogen of chronic periodontitis (Honda, 2011). *P. gingivalis* can produce outer membrane vesicles (OMVs) and release them to the surroundings (Grenier and Mayrand, 1987; Mayrand and Grenier, 1989), with the potential to affect distant tissues and organs such as brain, bone tissue, and others (Zhang et al., 2020). The OMVs play a potential role in systemic diseases related to *P. gingivalis* infection (Zhang et al., 2020; Okamura et al., 2021; Gong et al., 2022).

OMVs, secreted by Gram-negative bacteria, are spherical-bilayer structures with a diameter of 20-250 nm, which are composed of outer membrane proteins, lipopolysaccharides, phospholipids, DNA, and a part of the periplasm (Kulp and Kuehn, 2010). OMVs can package and carry the virulence factors of parent bacteria to various parts of the host (Kulp and Kuehn, 2010; Cecil et al., 2019). Virulence factors in OMVs provide advantages such as avoiding proteolytic degradation and facilitating long-distance communication (Bonnington and Kuehn, 2014; Jan, 2017; Zhang et al., 2020). Bacteria can interact with a broader range of their surroundings in this manner, which is especially advantageous for sessile bacteria like *P. gingivalis (* Tiku and Tan, 2021). Biogenesis of OMVs which is an energy-intensive process can be affected by multiple stress responses, such as environmental stresses and changes in nutrient composition of culture medium (Sartorio et al., 2021). Due to the complexity of OMVs’ structure and composition, an increasing number of researchers have conducted in-depth research on OMVs (Jan, 2017; Sharif et al., 2021).

*P. gingivalis* OMVs have been demonstrated to invade host cells such as human oral keratinocytes, gingival fibroblasts, and immune cells more swiftly than originating bacterial cells (Ho et al., 2015; Cecil et al., 2017; Fleetwood et al., 2017; Okamura et al., 2021; Zhang et al., 2022). *P. gingivalis* -derived OMVs can be internalized into epithelial cells via actin or lipid raft-mediated mechanisms, or directly linked with host cells by activating pattern recognition receptors (PRRs) (Zhang et al., 2020). Following their interaction with oral mucosal epithelial cells, *P. gingivalis* OMVs stimulated fibroblasts and epithelial cells to release IL-1β (interleukin 1β), IL-6 (interleukin 6), TNF-α (tumor necrosis factor-α), and other cytokines (Zhang et al., 2020). Along with the destruction of the epithelial barrier and the production of inflammatory cytokines (Furuta et al., 2009), *P. gingivalis* OMVs also promote cell dysfunction and activation, recruit immune cells (Nakao et al., 2014), drive macrophage metabolic remodeling, activate inflammasomes (Cecil et al., 2017; Fleetwood et al., 2017), and eventually result in periodontal tissue damage and alveolar resorption (Hajishengallis, 2014).

Damage and regeneration coexist in the progression of chronic periodontitis (Meyle and Chapple, 2015). Macrophages and periodontal ligament stem cells (PDLSCs) play an integral role in this process (Liu et al., 2019; Zhou and Graves, 2022). Macrophages are well-known pioneer cells in host innate immunity. They respond to antigens via phagocytosis, antigen presentation, and the production of IL-1β, IL-6, and TNF-α to induce inflammation and the recruitment of immune cells (Cecil et al., 2017; Deo et al., 2020). PDLSCs play a crucial function in maintaining homeostasis and initiating tissue regeneration, which is essential for periodontal tissue regeneration in inflammatory circumstances (Zhang et al., 2021). According to reports, purified *P. gingivalis* LPS can increase local inflammation of periodontal tissue and impair the multidirectional differentiation potential of PDLSCs (Bandow et al., 2010; Albiero et al., 2015). Furthermore, PDLSCs treated with *P. gingivalis* culture supernatant showed significant inhibition in cell proliferation, migration, and increased expression of inflammatory cytokines (Ramenzoni et al., 2019).

Pyroptosis has been implicated in various inflammatory diseases including periodontitis. K+ efflux, lysosomal damage, reactive oxygen species (ROS), and other external signals can activate the pyridine domain-containing NOD-like receptor family receptor 3 (NLRP3). The inflammasome regulates caspase-1 activation and promotes the maturation of Gasdermin D (GSDMD). Following that, cleaved GSDMD assembles on the plasma membrane, and the integrity of the plasma membrane is destroyed, resulting in the efflux of cellular contents. Pro-caspase-1 activation, on the other hand, stimulates the maturation and release of cytokines precursors pro-IL-1β and pro-IL-18, which increase the inflammatory effect. A study indicated that the concentration of ROS in vascular endothelial cells was increased after stimulation of *Helicobacter pylori* (*H. pylori*) OMVs (Wang et al., 2021). The same rise in ROS was observed in macrophages induced by *P. gingivalis* OMVs (Fleetwood et al., 2017). It is unclear whether NLRP3 was involved in the periodontal tissue damage induced by *P. gingivalis* OMVs.

Previous studies have provided some insight into the protein composition and subcellular localization of OMVs in parental bacteria cells (Zavan et al., 2019). However, there have been few investigations on the heterogeneity of OMVs’ physicochemical properties and biological functions. According to similar research, the quantity, size, and package contents of OMVs can vary depending on the bacterial development phases, OMVs from different growth stages having distinct biological attributes (Tashiro et al., 2010; Bonnington and Kuehn, 2014; Fleetwood et al., 2017; Gerritzen et al., 2017; Zavan et al., 2019; Bitto et al., 2021; Melo et al., 2021; Sharif et al., 2021; Wang et al., 2021). Sharif, E. et al. revealed that endotoxin-free *Escherichia coli* (*E. coli*) in the stationary phase produced more OMVs than other growth stages and that in terms of homogeneity, the OMVs produced in the pre-stationary phase were more uniform in size (Gui et al., 2016). The same phenomenon was also observed in the OMVs of *H. pylori*. *H. pylori* OMVs formed in different bacterial growth stages were substantially varied in diameter and proteins, as well as their ability to prompt cells to secrete IL-8 (Zavan et al., 2019).The findings above indicate that bacterial growth phases influence the physicochemical properties of OMVs (Bitto et al., 2021).

OMVs are formed throughout the growth cycle of *P. gingivalis*. Nonetheless, the majority of research focuses on *P. gingivalis* OMVs collected in the late logarithmic growth stage, while the pathogenic effects of OMVs produced in other growth stages are seldom studied (Deng et al., 2022). Here we carried out a comparative study on the physical properties, protein composition, pathogenicity, and immune response of *P. gingivalis* OMVs collected at different growth stages, aiming to provide certain insights for the in-depth exploration of *P. gingivalis* OMVs.

## 2 Materials and methods

### 2.1 Culture of *Porphyromonas gingivalis* (W83) and detection of growth curves

*P. gingivalis* (W83) was cultured in brain-heart infusion (OXOID, Basingstoke, Britain) containing 5mg/L hemin, and 0.5mg/L vitamin K in an anaerobic environment (80% N_2_ -10% CO_2_ -10% H_2_, 101KPa). *P. gingivalis* (W83) liquid cultures were inoculated at an optical density (OD _600nm_) of 0.08. And the value of OD _600nm_ was measured every 6 hours.

### 2.2 Isolation of Porphyromonas gingivalis OMVs

*P. gingivalis* OMVs were isolated by an established protocol (Zhang et al., 2020; Okamura et al., 2021; Gong et al., 2022). Briefly, we first determined the growth stage of *P. gingivalis* via the growth curve, and then selected the pre-log, late-log, and stationary stages as the time nodes for OMVs extraction. *P. gingivalis* (W83) was cultured in an anaerobic environment to pre-log(18 hours), late-log(30 hours), and stationary growth stages(54 hours). After the bacterial culture medium was collected and centrifuged (4°C, 8, 000g, 40 minutes), the supernatants were filtered with a 0.22-μm syringe filter., then concentrated with an 50ml Ultra-15 Centrifugal Filter Device with a membrane nominal molecular weight limit (NMWL) 100 KDa (Amicon, Merck, USA) at 4°C, 4, 000 rpm for 15 minutes. The supernatant was ultracentrifuged at 100, 000g for 120 minutes at 4°C, and resuspended the pellet in PBS. The OMVs were stored at -80°C. OMVs were quantified by protein concentrations using a BCA protein assay kit (Beyotime, Shanghai, China).

### 2.3 Identification of *Porphyromonas gingivalis* OMVs

The diameters and the numbers of OMVs produced by *P. gingivalis* (W83) in the pre-log, late-log, and stationary stages were measured by nanoparticle tracking analysis (NTA). The specific steps are as follows: Firstly, we took freshly extracted OMVs 50μl preparation for testing and cleaned the sample cell with deionized water; Then, the instrument was calibrated using polystyrene microspheres (110nm) and cleaned the sample pool with 1 × PBS buffer (Biological Industries, Israel); Finally, we used 1 × PBS buffer to dilute the sample for testing. Repeat testing three times for each sample.

The morphology of *P. gingivalis* OMVs were observed via transmission electron microscopy (TEM). Freshly extracted OMVs was diluted 500 times and transported on ice; Secondly, we dropped OMVs diluted to a suitable magnification onto the electron microscope copper mesh grid and waited for 10 minutes; Thirdly, we added 2% uranyl acetate dropwise to the copper net for 3 minutes, and cleaned twice with deionized water; Finally, take photos under a transmission microscope.

### 2.4 SDS-PAGE

OMVs were incubated in a sodium dodecyl sulfate (SDS) loading buffer containing 5% 2-mercaptoethanol at 100°C for 5 minutes and then they were separated using polyacrylamide gel electrophoresis (PAGE). The separated OMVs proteins were stained with 0.25% Coomassie brilliant blue, then rinsed for 30 minutes with PBS, until the proteins bands were clear.

### 2.5 Isolation and culture of human periodontal ligament stem cells (PDLSCs)

PDLSCs were isolated from healthy periodontal ligaments of premolars or third molars extracted from young donors (n=10, aged 18-25 years) undergoing orthodontic treatment in Surgical outpatient Department of Stomatological Hospital affiliated to Chongqing Medical University. This study was approved by the Ethics Committee of the affiliated Stomatological Hospital of Chongqing Medical University (CQHS-REC-2023 (LSNo.008)), and informed consent was obtained from all volunteers. Periodontal ligament tissue was digested in collagenase I solution with concentration of 3mg/ml collagenase I solution at 37°C for 30 minutes (Sigma, St. Louis, MO, USA), and then centrifuged at 300*g* for 2 minutes. Afterwards, the tissue block precipitation was resuspended with α-MEM (Gibco, Grand Island, USA) supplemented with 10% fetal bovine serum (LONSERA, Shanghai, China), 100 U/ml penicillin, and 100mg/ml streptomycin (Hyclone, State of Utah, USA) at 37°C in a humidified atmosphere (20% O_2_, 5% CO_2_) at 37°C. All experiments in this study were performed using passage 3 to passage 5 cells.

### 2.6 Cell culture

PDLSCs grown in α-MEM (Gibco, Grand Island, USA) and macrophages grown in DMEM (Gibco, Grand Island, USA) were supplemented with 10% fetal bovine serum (LONSERA, Shanghai, China), 100 U/ml penicillin, and 100mg/ml streptomycin (Hyclone, State of Utah, USA) at 37°C in a humidified atmosphere (20% O_2_, 5% CO_2_) at 37°C.

### 2.7 Co-culture of *Porphyromonas gingivalis* OMVs with macrophages

Macrophages (RAW264.7) was obtained from the Cell Bank of the Chinese Academy of Sciences (SCSP-5036, Shanghai, China). RAW264.7 were seeded into 6-well plates (3×10^6^ cells/well) and cultured overnight. Then cells were treated with *P. gingivalis* OMVs (10μg/ml protein concentration) in different growth stages (pre-log, late-log, and stationary stage), respectively. Cells samples were collected after 24 hours of stimulation with *P. gingivalis* OMVs.

### 2.8 Flow cytometry

The expression of CD29, CD31, CD45, CD90, and CD105 in PDLSCs and CD86, F4/80 in RAW264.7 were analyzed via Flow Cytometry. Isotype controls were run in parallel. Flow cytometry was performed using a Cyto FLEX flow cytometer (BD Biosciences, State of New Jersey, USA). Experimental results were analyzed using FLOW JO (BD Biosciences, State of New Jersey, USA).

### 2.9 Co-culture of *Porphyromonas gingivalis* OMVs with PDLSCs

PDLSCs were seeded into 6-well plates (3×10^6^ cells/well) and cultured overnight. Then cells were treated with *P. gingivalis* OMVs (10μg/ml protein concentration) in different growth stages (pre-log, late-log, and stationary stage), respectively. Cells samples were collected after 24 hours of stimulation with *P. gingivalis* OMVs.y.

### 2.10 Cell internalization experiment

RAW264.7 and PDLSCs are cultured with OMVs derived from *P. gingivalis* labeled by PKH26(Sigma-Aldrich, St Louis, USA), and the specific steps are as follows. Firstly, inoculate RAW264.7 and PDLSCs into 6-well plates (10^6^ cells/well) in advance; Secondly, 100μL PBS was used to resuspend OMVs, and the solution was mixed with 500μL Diluent C; Add 2μL PKH26 and 500μL Diluent C; Mix the above two solutions and incubate for 4 minutes; Terminate the staining step with 2mL 0.5% BSA and incubate for 5 minutes. Re-extract the stained OMVs according to the process.

Then we incubated the stained OMVs with RAW264.7 and PDLSCs for 1 and 4 hours. Subsequently, the cells were washed 3 times with PBS, and then fixed with 4% PFA for 10 minutes at room temperature. The cells were washed with PBS again and incubated with diluted Actin-Tracker Green(Beyotime, Shanghai, China) for 1 hours. Wash with immunostaining detergent (Beyotime, Shanghai, China) for 2-4 times, and 5 minutes each time. Observation under fluorescence microscope.

### 2.11 Evaluations of PDLSCs migration and proliferation

Scratch wound assays were conducted to assess the effects of *P. gingivalis* OMVs on migration, as previously described (Izui et al., 2021). Confluent cells were slightly scraped across the center of the well with a sharp point. Afterward, serum-free medium mixed with 10μg/ml protein concentration of *P. gingivalis* OMVs of each stage were added. Then the plate was incubated in a humidified environment at 37°C, 95% air, and 5% CO_2_. We selected a total of 3 fields of view for image acquisition and pictures were taken at 0, 24, and 48 hours. Then we used image J to measure the scratch area and quantified the scratch wound assays by calculating the scratch healing rate for three fields of view. Area detection method (scratch distance measurement is an equivalent measurement). The average scratch width = scratch gap area/length. Cell migration rate= (0 h scratch width - scratch width after culture)/0 h scratch width × 100%.

To explore the effects of *P. gingivalis* OMVs on the proliferation of cultured cells, the medium containing 5 × 10^3^ PDLSCs/ml/well was pipetted into 96-well culture plates and cultured in a humidified environment at 37°C, 95% air, and 5% CO_2_. After the cells adhered, 10μg/ml of pre-log, late-log, and stationary OMVs were added to each group respectively. The absorbance at OD _450nm_ was detected at 24 hours, 48 hours, and 72 hours thereafter. Cell proliferation rate=(absorption value of experimental group - blank control absorption value)/(absorption value of control group - blank control absorption value) × 100%.

### 2.12 Alkaline phosphatase staining and activity assay

ALP staining: PDLSCs were seeded (4×10^4^ cells/well) overnight. Then replaced them with osteogenic induction medium (β-glycerophosphate sodium 10mM, Dexamethasone 10^-4^mM, Vitamin C 50μg/ml) with 10μg/ml OMVs of different growth stages the next day. Replace the above medium every two days. On the seventh day of processing, collect cell samples for ALP staining as previously described (Park et al., 2021). In short, Cells were fixed, and treated with the ALP reaction solution (Beyotime, Shanghai, China) for 1 hour at 37°C.

ALP activity assay: The previously established experimental methods were used (Bruedigam et al., 2011). Briefly, ALP activity was assessed using a microplate reader (PerkinElmer, Massachusetts, USA) after converting para-nitro phenyl phosphate (pNPP) to paranitrophenol for 10 minutes at 37°C.

### 2.13 Establishment of periodontitis model in rats

In this study, 36 wild-ty pe Sprague Dawley rats (6 weeks old, male) were used, which were purchased from the Animal Experiment Research Center of Chongqing Medical University. All animal care and research protocols were approved by the Experimental Animal Ethics Committee of Chongqing Medical University (CQHS-REC-2023 (LSNo.008)). All research involving animals was conducted following relevant ethical regulations. After three-day acclimatization, to inhibit endogenous bacteria that are not conducive to the colonization and growth of *P. gingivalis*, another five -day antibiotic treatment (amikacin sulfate 3mg/ml, Solarbio, Beijing, China) were given through drinking water, and the antibiotic solution was applied to the oral cavity of the rats. Ligation-induced periodontitis was constructed as previously described (Park et al., 2017; Pereira et al., 2022). Briefly, rats were anesthetized with isoflurane and their oral cavity was disinfected (75% ethanol), and a 2-0 ligation wire was ligated around the right upper first molar. All knots were placed on the palatal side. Once the ligation was finished, rats were then placed in a warmer environment until they awoke, and were given free access to food and water.

Rats were divided into 6 groups: unligation group, PBS +ligation group, pre-log OMVs +ligation group, late-log OMVs +ligation group, stationary OMVs +ligation group, and *P. gingivalis* +ligation group. Except for the unligation group, each group was injected with 10μl PBS, pre-log OMVs, late-log OMVs, stationary OMVs, and *P. gingivalis* through the gingival sulcus around the ligature wire every two days. All OMVs are given a concentration of 10mg/ml, while *P. gingivalis* are given a concentration of 10^9/^CFU. After 4 weeks of ligation, the rats were euthanized. Periodontal tissues were collected for analysis to detect the pathophysiological changes of periodontitis.

### 2.14 Micro CT

Paraformaldehyde-fixed rats’ maxillary specimens were scanned with micro-CT (SCANCO Medical AG, Switzerland), and 3D reconstructions were performed using Photoshop to measure the distance from the cementoenamel junction to the alveolar crest (CEJ-ABC). The Sky Scan Data viewer was used to analyze the changes in the alveolar bone in the distal region of the maxillary first molars.

### 2.15 H&E staining

The paraffin-embedded alveolar bone tissue was sectioned into 6 μm thick sections and then stained with H&E to observe the changes in the alveolar bone, periodontal ligament, and gingival connective tissue. Images were acquired by microscopy (Olympus, Tokyo, Japan).

### 2.16 Tartrate acid phosphatase staining (TRAP)

To identify osteoclasts, tissue sections were stained with a TRAP kit (Servicebio, Wuhan, China). Under a microscope (Olympus, Tokyo, Japan), TRAP -stained histological tissue sections were inspected, and photographs of designated locations were taken.

### 2.17 RNA extraction and RT-qPCR analysis

Total RNA was extracted using the RNAeasy™ Plus Animal RNA Extraction Kit (Beyotime, Shanghai, China) and was subjected to reverse transcription into cDNA via the TAKARA Reverse Transcription Kit (Takara, Kyoto, Japan). Quantitative real-time PCR was performed in the ABI Prism 7500 Real-Time PCR System (Bio-Rad, Hercules, USA) with the SYBR Green PCR master mix reagent (Takara, Tokyo, Japan) according to the manufacturer’s instructions. The relative quantitative value of each gene was calculated by the 2-ΔΔCt method. GAPDH was used as an internal reference.

### 2.18 Western blot

Western blot analysis was performed to measure protein expression levels in PDLSCs. The collected cell samples were lysed in RIPA lysis buffer (Beyotime, Shanghai, China) supplemented with protease and phosphatase inhibitor cocktail (ComWin Biotech, Beijing, China). The samples were centrifuged at 4°C, 7, 500 rpm, 5 minutes, then the supernatant was collected. Subsequently, the samples were incubated with 5× loading buffer (Beyotime, Shanghai, China) for 5 minutes at 100°C. The incubated protein samples were separated on 10% tris-glycine SDS-PAGE and transferred to PTM polyvinylidene fluoride (PVDF) membranes (Millipore, Burlington, MA, United States). To block nonspecific background, the membranes were blocked with 5% nonfat milk in Tris-buffered saline containing 0.1% Tween-20 (TBST) for 2 hours at room temperature. The target proteins were immunoblotted overnight at 4°C with the following primary antibodies: GAPDH (1:2, 000, Abcam, Cambridge, UK), NF-κB p65 (1:1, 000, Abcam), NLRP3(1:1, 000, Abcam), IL1β (1:1, 000, Abcam), caspase1(1:1, 000, Abcam), OSX (1:1, 000, Abcam), Runx2(1:1, 000, Abcam), ALP (1:1, 000, Abcam). Anti-mouse IgG or anti-rabbit IgG secondary antibodies (Cell Signaling Technologies, Boston, USA) were incubated with the target proteins for 2 hours at room temperature. The blots were photographed using the Bio-Rad Imager and ECL Western blotting substrate (Beyotime, Shanghai, China). The relative intensity of each protein was quantified by ImageJ software.

### 2.19 Protein mass spectrometry iTRAQ

The extracted OMVs produced by *P. gingivalis* (W83) at different periods were subjected to protein-relative quantitative analysis (Jinkairui, Wuhan, China). The details of the proteomic procedure and data analysis are included in the **Supplementary Material**.

### 2.20 Data analysis

All statistical analyses were performed using Prism software (GraphPad Software, San Diego, CA, USA). ANOVA was used for all statistical analyses. Data were expressed as means ± SEM, and differences were considered significant when p < 0.05.

### 2.21 Ethics approval and consent to participate

All protocols were approved by the by the Experimental Animal Ethics Committee of Chongqing Medical University (CQHS-REC-2023(LSNO.008)). Before enrolment, each participant gave their informed permission.

## 3 Results

### 3.1 The effect of growth stages on the characterization of *Porphyromonas gingivalis* OMVs

**Figure 1:**
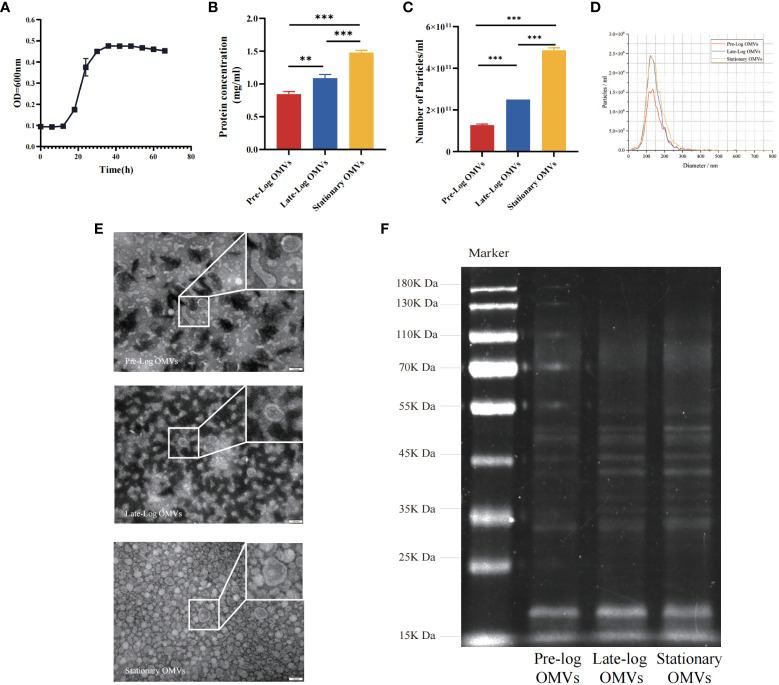
Isolation, identification and preliminary comparison of *P. gingivalis* OMVs in different growth stages. **(A)** Determination of growth curve of P. gingivalis (W83). The bicinchonininc acid (BCA) assay **(B)** and nanoparticle tracking analysis (NTA) **(C)** were used to quantify the *P. gingivalis* OMVs in different growth stages. **(D)** The mean particles size of all groups distribution was evaluated via NTA (Red: Pre-Log OMVs; Blue: Late-Log OMVs; Yellow: Stationary OMVs). **(E)** Transmission electron microscope (TEM) of *P. gingivalis* OMVs in different growth stages. (Scale bar = 100nm) **(F)** Preliminary comparison of OMVs proteins in different growth stages by SDS-PAGE found differences in protein bands ranging from 25 to 75 KDa. (n=3; **P < 0.01, ***P < 0.001).

Firstly, we cultured *P. gingivalis* (W83, in anaerobic conditions and studied its growth curve. *P. gingivalis* (W83) reached the pre-log phase, late-log phase, and stationary growth phase at 18 hours, 30 hours, and 54 hours, respectively (**Figure 1A**). *P. gingivalis* OMVs were isolated from cultures at different growth stages by ultrafiltration and ultracentrifugation. Concerning the yield of OMVs, an average of 1.27×10^11^, 2.50×10^11^, and 4.87×10^11^ OMVs per 100 ml of culture supernatant was obtained from the pre-log, late-log, and stationary bacterial cultures, respectively. Meanwhile, the protein concentration of pre-log, late-log, and stationary *P. gingivalis* OMVs was detected using a BCA assay. The results indicated that the protein yield of *P. gingivalis* OMVs increased with culture time and peaked at the stationary stage (**Figures 1B, C**). According to NTA, there was no significant difference in the size distribution of *P. gingivalis* OMVs in different growth phases, with mean diameters of 100 nm at all stages (**Figure 1D**). In terms of morphology, pre-log OMVs weren’t as homogenous as late-log and stationary OMVs, with certain anomalies like long strips and ovals observed by TEM (**Figure 1E**). SDS-PAGE showed that there were differences in the density of protein bands from 25KDa to 75KDa in the OMVs of *P. gingivalis* collected at different growth stages (**Figure 1F**).

As demonstrated by the above results we found that the morphology of *P. gingivalis* OMVs in the early growth stage (pre-log OMVs) was more diverse than that of the other two stages. With the prolongation of the bacterial culture time, the yield of *P. gingivalis* OMVs increased, and there were clear variations in protein composition. More research will be taken to determine whether these changes affect the biological function of *P. gingivalis* OMVs.

### 3.2 *Porphyromonas gingivalis* OMVs contain different protein profiles during various growth stages

**Figure 2:**
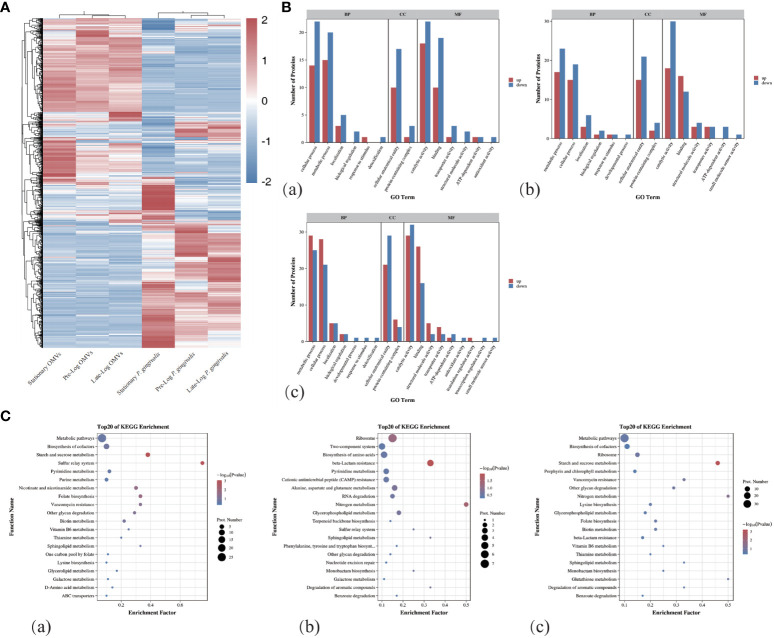
Protein mass spectrometry analysis of OMVs produced by *P. gingivalis* in different growth stages. **(A)**The hierarchical clustering analysis of the proteins of OMVs and bacteria in different growth stages showed that there were obvious differences in the composition of proteins between OMVs and bacteria in different stages. **(B)** GO Term analysis showed that compared with Pre-Log, Late-Log and Stationary OMVs had significantly more up-regulated than down-regulated proteins in cellular process, metabolic process, cellular anatomical entity, and catalytic activity. **(C)** Bubble plots of the top 20 rankings for significant enrichment analysis in the comparative group GO functional enrichment results. ((a) Pre-Log OMVs vs Late-Log OMVs, (b) Pre-Log OMVs vs Stationary OMVs, (c) Late-Log OMVs vs Stationary OMVs).

As shown in **Figure 1F**, we performed a SDS-PAGE analysis of the protein component of *P. gingivalis* OMVs in different growth stages and discovered heterogeneity among them. The differential expression of protein identification and quantitation was performed via LC-MS/MS (liquid chromatograph mass spectrometer). **Table 1** showed the number of the differential expression of the protein of *P. gingivalis* OMVs and *P. gingivalis* (W83) in each growth stage. A total of 1,183 proteins were identified in *P. gingivalis* (W83) and *P. gingivalis* OMVs(supplement materials). For differential expression, 67 differential proteins were found between the pre-log and late-log stages. 120 differential proteins were found between the pre-log and stationary stages, and 137 differential proteins were found between the late-log and stationary stages. The hierarchical cluster analysis heat map results showed the same trend as those of SDS-PAGE (**Figure 2A**). Protein subcellular localization analysis revealed (**Supplementary Figure 1**) that the proteins of *P. gingivalis* OMVs include proteins in the cytoplasm and inner membrane, outer membrane, and periplasm. The sources of the protein were not significantly different (**Supplementary Figure 1**). The assembly components of *P. gingivalis* OMVs appear to be rather constant during each cycle of bacterial growth.

**Table 1:** Number of significant differences between pairwise samples.

Compared name	Total quant	Up-regulated	Total diff	Down-regulated
Pre-Log *P. gingivali*s OMVs vs Pre-Log *P. gingivalis*	1183	326	628	302
Late-Log *P. gingivali*s OMVs vs Late-Log *P. gingivalis*	1183	339	622	283
Stationary *P. gingivali*s OMVs vs Stationary *P. gingivalis*	1183	370	710	340
Pre-Log *P. gingivali*s OMVs vs Late-Log *P. gingivalis* OMVs	1183	29	67	38
Pre-Log *P. gingivali*s OMVs vs Stationary *P. gingivalis* OMVs	1183	47	120	73
Late-Log *P. gingivali*s OMVs vs Stationary *P. gingivalis* OMVs	1183	52	139	87

In the process of Gene Ontology (GO) annotation (**Figure 2B**), late-log OMVs (**Figure 2B**a) and stationary OMVs (**Figure 2B**b) were significantly up-regulated in biological process, cellular composition and molecular function compared with pre-log OMVs——including cellular process, metabolic process in biological process, the cellular anatomical entity in cell composition, catalytic activity and binding in molecular function. Compared with late-log OMVs (**Figure 2B**c), pre-log OMVs showed more differential protein upregulation in the cellular process, metabolic process, and binding.

The Kyoto Encyclopedia of Genes and Genomes (KEGG) enrichment analysis (**Figure 2C**) showed significantly altered metabolic pathways in the protein composition of *P. gingivalis* OMVs in different growth stages. From the comparison among pre-log (**Figure 2C**a), late-log (**Figure 2C**b), and stationary OMVs, it can be seen that pre-log OMVs have more up-regulated proteins related to starch and sucrose metabolism and glycerol phosphate metabolism, which may be related to the rapid proliferation of bacteria in the pre-log stage. Compared with pre-log OMVs, late-log OMVs and stationary OMVs expressed more resistance-related proteins, including vancomycin resistance, β-lactam resistance, cationic antimicrobial peptide resistance, and so on. (**Figure 2C**c).

In summary, compared with the other two groups, OMVs collected in pre-log cultures contained more proteins related to bacterial metabolism, which was likely due to the rapid proliferation of bacteria. Late-log OMVs and stationary OMVs contained more resistance-related proteins than pre-log OMVs. Further research is required to determine whether these distinctions will lead to subsequent differential host responses.

### 3.3 *Porphyromonas gingivalis* OMVs entered macrophages more quickly than PDLSCs independent of growth stages

**Figure 3:**
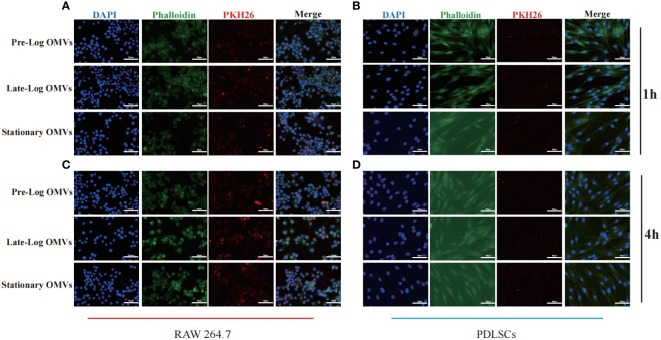
Cell internalization experiments of *P. gingivalis* OMVs. 1h of co-incubating with RAW264.7 **(A)** and PDLSCs **(B)** and 4h of co-incubating with RAW264.7 **(C)** and PDLSCs **(D)**. (Scale bar = 100nm) (DAPI: cell nuclei; Red: OMVs; Green: Cytoskeleton).

To detect the time when *P. gingivalis* OMVs enter into cells, PDLSCs and macrophages were treated with PKH26-labeled *P. gingivalis* OMVs in different growth stages at a concentration of 10μg/ml and viewed under a microscope after incubation for 1 hour and 4 hours, respectively. After 1 hour of stimulation, there are more OMVs observed in macrophages than those in PDLSCs (**Figures 3A, B**). After 4 hours of stimulation, both macrophages and PDLSCs have a considerable amount of OMVs (**Figures 3C, D**).

The foregoing experimental results proved that there was no significant difference in the internalization time of *P. gingivalis* OMVs in different growth stages in cells of the same type. Different cell types responded to *P. gingivalis* OMVs at different rates. Macrophages, as first-line immune response cells, internalized *P. gingivalis* OMVs more quickly than PDLSCs.

### 3.4 The late-log OMVs and stationary OMVs were more effective in promoting macrophages toward the proinflammatory M1-like phenotype than pre-log OMVs

**Figure 4:**
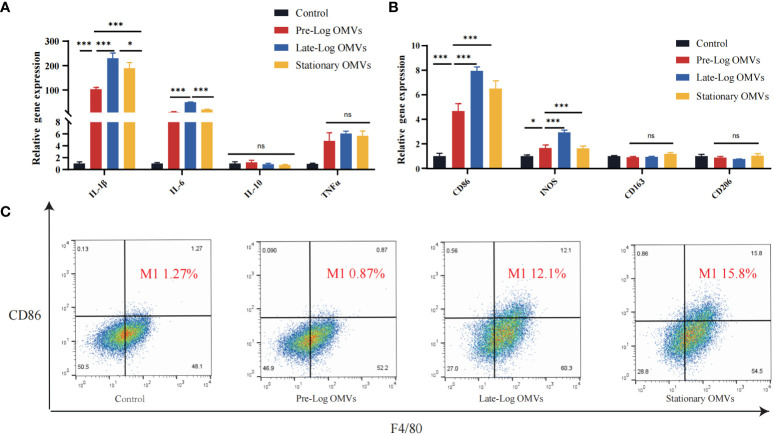
*P.* gingivalis OMVs in different growth stages can stimulate the expression of inflammatory cytokines-related genes and increase differentiation towards the pro-inflammatory phenotype (M1) in RAW264.7 after 24 hours of treatment. **(A)** *P. gingivalis* OMVs in different growth stages can induce the expression of inflammation-related genes in RAW264.7. RT-qPCR **(B)** and flow cytometry analysis **(C)** demonstrated the effect of *P. gingivalis* OMV on macrophage polarization. (FITC: F4/80; PE: CD86). (n=3; *P < 0.05, ***P < 0.001). The symbol ns means “no significance”.

Since we discovered that *P. gingivalis* OMVs proteins in different growth stages were differentially expressed in biological processes, cellular composition, and molecular functions (**Figure 2B**), the effect of protein composition on OMVs biological function were further investigated, we stimulated macrophages with 10 μg/ml of *P. gingivalis* OMVs for 24 hours. The results of RT-qPCR showed that compared with pre-log OMVs, the mRNA expression of inflammatory cytokines, such as IL-1β, IL-6, and TNF-α, was significantly increased in the late-log and stationary OMVs groups (**Figure 4A**). We further investigated the effect of *P. gingivalis* OMVs in different growth stages on macrophage polarization via examining the expression of related genes. The late-log OMVs and stationary OMVs groups significantly enhanced the expression of M1 macrophage biomarkers (CD86, INOS) compared to the pre-log OMVs group(P<0.001) (**Figure 4B**). Regarding the genes related to M2 macrophage biomarkers (CD206, CD106), there was no significant difference between each group and the control group.

Flow cytometry results confirmed the RT -qPCR results (**Figure 4C**). We chose CD86 as the M1 macrophages biomarker and F4/80 as the M0 macrophages biomarkers. Macrophages stimulated by late-log and stationary OMVs possessed 12.1% and 15.8% M1 macrophages, respectively, while macrophages stimulated by pre-log OMVs had just 0.87% M1 macrophages. Taken together, these findings implied that late-log OMVs and stationary OMVs more significantly promoted macrophages toward the proinflammatory M1-like phenotype and increased inflammatory cytokines mRNA expression than pre-log OMVs.

### 3.5 The late-log OMVs and stationary OMVs exhibited more cytotoxicity on PDLSCs compared to pre-log OMVs

**Figure 5:**
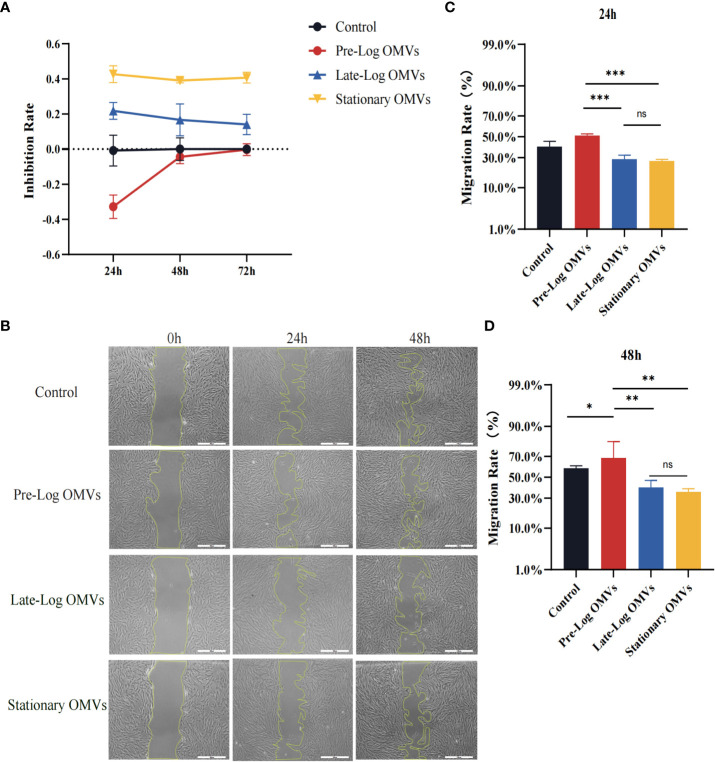
*P.* gingivalis OMVs in different growth stages inhibited the proliferation and migration of PDLSCs to different degrees. **(A)** The *P. gingivalis* OMVs in different growth stages inhibited the proliferation of PDLSCs from 24h to 72h in CCK-8 experiments. **(B)** Scratch test results showed that *P. gingivalis* OMVs in different growth stages inhibited the migration of PDLSCs at 24h and 48h. (Scale bar = 500nm). **(C, D)** Quantitative analysis of scratch test results. (n=3; *P < 0.05, **P < 0.01, ***P < 0.001). The symbol ns means “no significance”.

Earlier in this study, it had been demonstrated that PDLSCs showed MSC-like (Mesenchymal stem cells-like) characteristics (**Supplementary Figure 2**). To investigate the influence of *P. gingivalis* OMVs on PDLSCs proliferation, PDLSCs were treated with 10 μg/ml *P. gingivalis* OMVs in different growth stages for 24 hours and 48 hours respectively (**Figure 5A**) and detected with CCK-8 assay. After 24 hours, pre-log OMVs marginally increased cell proliferation activity, whereas late-log and stationary OMVs considerably reduced cell proliferation activity. Meanwhile, late-log OMVs inhibited cell proliferation the most. The same trend was observed after *P. gingivalis* OMVs treating PDLSCs for 48 hours and 72 hours (**Figure 5A**).

The findings of the scratch wound experiment substantiated the cytotoxic effects of OMVs on PDLSCs (**Figure 5B**). Likewise, PDLSCs were treated with 10 μg/ml of *P. gingivalis* OMVs in different growth stages, and photographs were taken after 24 hours and 48 hours. At 24 hours, wound closure processes were clearly observed for the control and pre-log OMVs groups, whereas it merely started in late-log and stationary OMVs groups (**Figures 4B, C**). After another 24 hours, when the surrounding cells of the control group recovered to 58.99%, the migration rate of late-log and stationary groups remained at about 39.73% and 35.47%, respectively (**Figures 4B–D**). It is worth noting that the addition of pre-log OMVs slightly promoted the healing of wound with a migration rate of 68.02% (**Figures 4C, D**).

### 3.6 The late-log OMVs and stationary OMVs significantly inhibited the osteogenesis of PDLSCs

**Figure 6:**
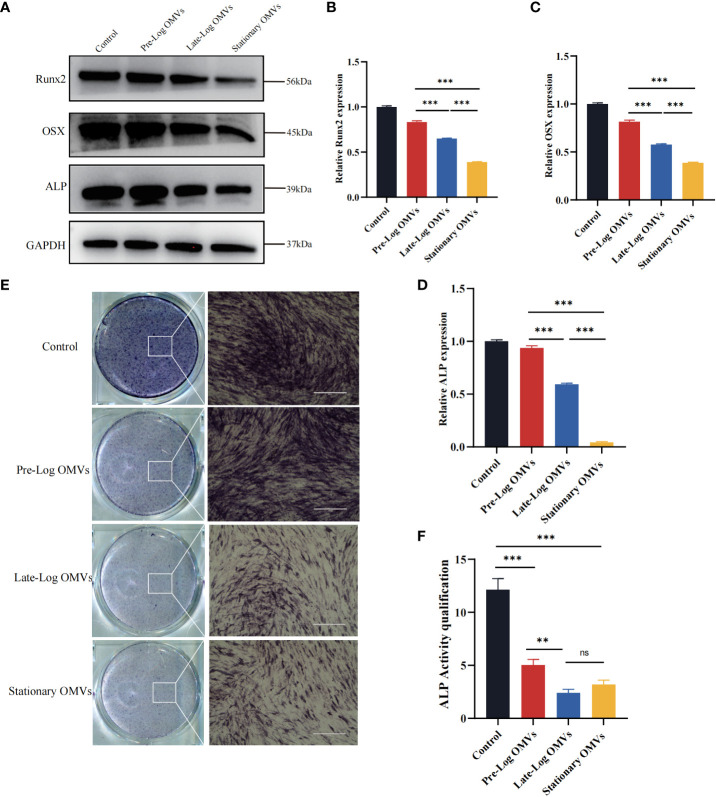
*P*. gingivalis OMVs in different growth stages inhibited osteogenesis of PDLSCs. **(A)** Western blot results showed that *P.* gingivalis OMVs in different growth stages varied to influence on osteogenesis. The late-log and stationary OMVs significantly inhibited the expression of osteogenesis-related proteins, while the effect of the pre-log OMVs was slightly smaller. **(B–D)** The western blot relative expression of Runx2, OSX, ALP. **(E)** The results of alkaline phosphatase (ALP) cells staining were consistent with the previous results. (Scale bar = 400nm). **(F)** Quantitative analysis of alkaline phosphatase activity. (n=3; **P < 0.01, ***P < 0.001). The symbol ns means “no significance”.

The osteogenic differentiation of PDLSCs was examined in our experiments. After stimulating PDLSCs with10 μg/ml of *P. gingivalis* OMVs for 7 days, the osteogenesis-associated proteins such as ALP (Alkaline phosphatase), Runx2, and OSX (Osterix) in the late-log and stationary OMVs groups were considerably reduced, whereas there was no significant difference between the pre-log OMVs and control groups (**Figures 6A–D**). The findings of ALP staining and quantification exhibited the same trend (**Figures 6E, F**).

Altogether, both late-log OMVs and stationary OMVs had strong inhibitory effects on the osteogenesis of PDLSCs, while pre-log OMVs’ inhibition didn’t seem that obvious. We hypothesize that late-log OMVs and stationary OMVs can cause periodontal damage by increasing inflammatory cytokines release and suppressing PDLSCs proliferation, migration, and osteogenic differentiation.

### 3.7 Variations in growth stages of *Porphyromonas gingivalis* OMVs affected the activation of NLRP3 inflammasome

**Figure 7:**
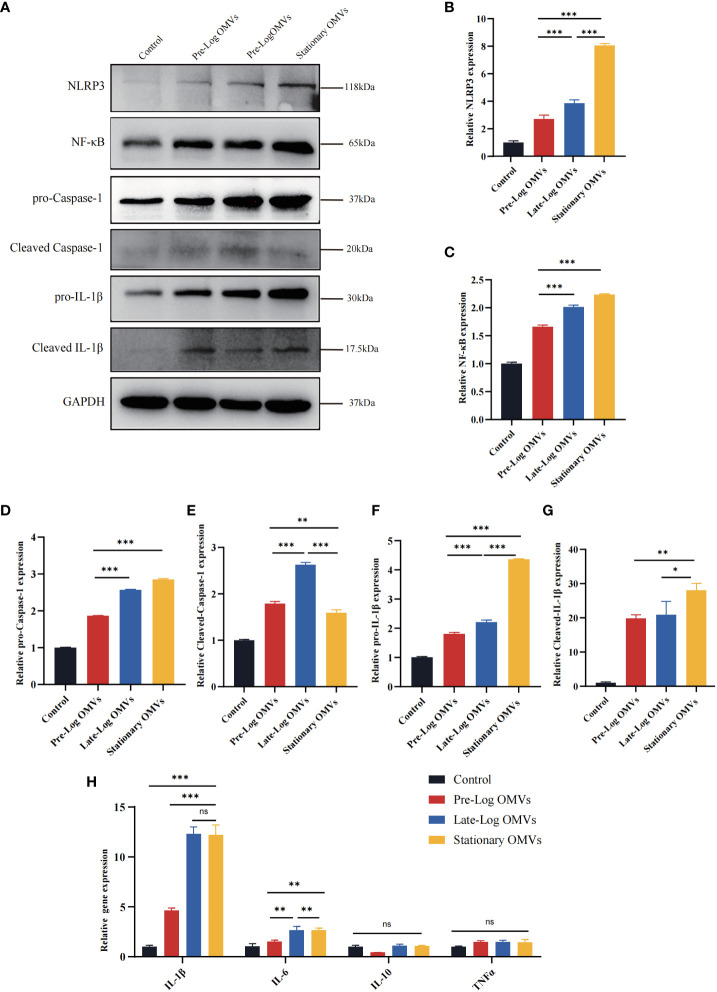
*P*. gingivalis OMVs in different growth stages can activate the NLRP3 inflammasome and induce the expression of inflammation-related genes in PDLSCs to different degrees. **(A)** Western blot results showed the expression of proteins associated with NLRP3/IL1β signaling pathway were significantly elevated. **(B–G)** The western blot relative expression. **(H)** After 24h of treatment, *P. gingivalis* OMVs in different growth stages can induce the expression of inflammation-related genes in PDLSCs and the effects of late-log and stationary OMVs were more significant. (n=3; *P < 0.05, **P < 0.01, ***P < 0.001.). The symbol ns means “no significance”.

Existing studies indicate that the NLRP3/IL-1β signaling pathway played a crucial role in the occurrence and development of periodontitis (Cheat et al., 2022; Zhao et al., 2022). As a result, we detected the NLRP3 inflammasome-related proteins in PDLSCs (**Figures 7A–G**). The expressions of NLRP3, pro-caspase-1, pro-IL-1β, cleaved caspase-1, and cleaved IL-1β were the highest in the stationary OMVs group and lowest in the pre-log OMVs group. Additionally, the expression of NF-κB was also considerably increased in the OMVs group and somewhat higher in the stationary OMVs group than in the other two groups (**Figure 7C**). The expression of inflammatory cytokines-related genes was then measured 24 hours after *P. gingivalis* OMVs treated PDLSCs in each group (**Figure 7F**). In comparison to pre-log OMVs, the mRNA expression of inflammatory cytokines such as IL-1β and IL-6 was dramatically elevated in the late-log and stationary OMVs groups, while gene expression of the anti-inflammatory factor (IL-10) had not significantly changed. Meanwhile, the pre-log OMVs group promoted less (or even suppressed) cytokine production compared with the other groups (**Figure 7H**). When comparing the expression levels of cytokines produced by macrophages (**Figure 4A**) and PDLSCs (**Figure 7H**), macrophages were more responsive than PDLSCs, producing larger amounts of inflammation-related cytokines.

Overall, compared to pre-log OMVs, the late-log and stationary OMVs significantly increased NLRP3 inflammasome activation in PDLSCs, eliciting a variety of immunological and inflammatory responses in host cells.

### 3.8 The late-log OMVs and stationary OMVs caused serious periodontal destruction *in vivo*

**Figure 8:**
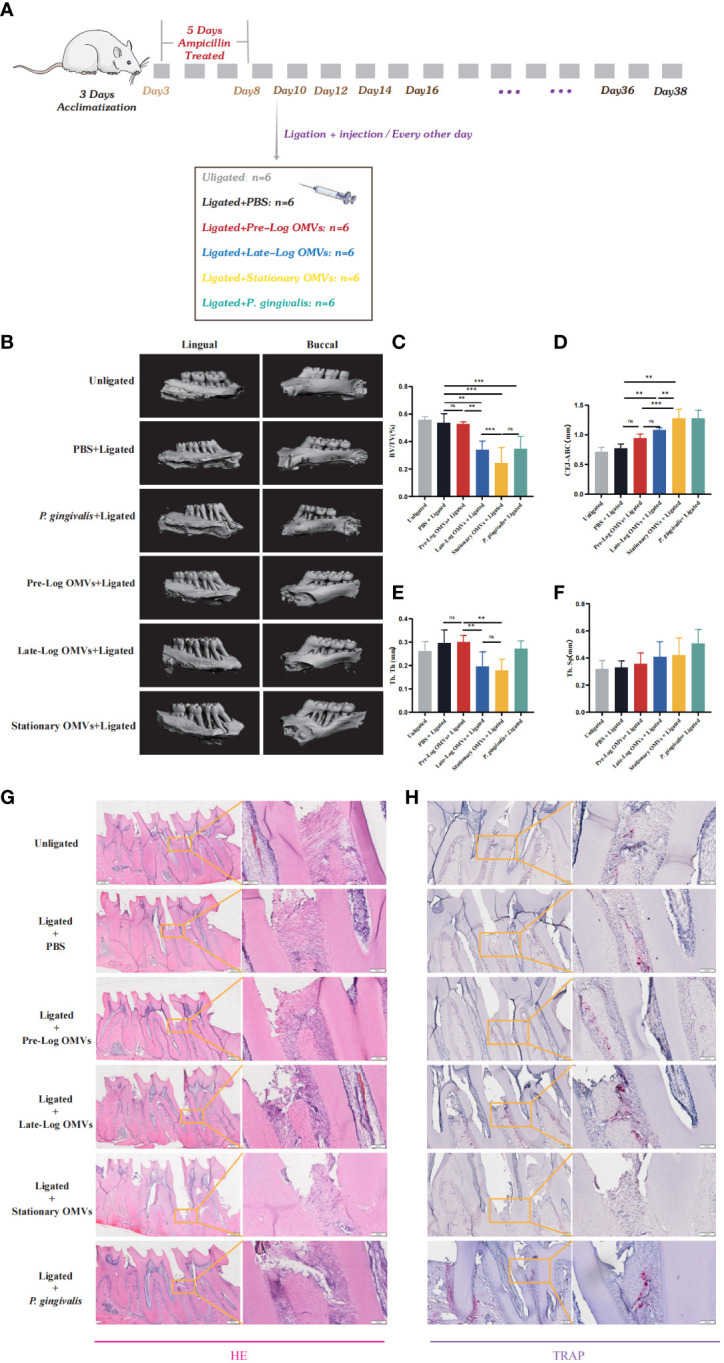
*P.* gingivalis OMVs in different growth stages can cause different degrees of periodontitis in rats. **(A)** The periodontitis mouse model was constructed. **(B)** After ligation and local injection for 1 months, the alveolar bone of rats was detected by micro-CT. The results showed that the rat alveolar bone had different degrees of attachment loss. Micro- CT showed that there were significant differences in BV/TV **(C)**, CEJ-ABC **(D)**, Tb. Th **(E)**, and Tb. Sp **(F)** of alveolar bone tissue in each group of rats. **(G)** Staining of alveolar bone with HE. **(H)** Staining of alveolar bone with TRAP. (n=6; * p<0.05). CEJ, cement-enamel junction; ABC, alveolar bone crest; BV/TV, bone volume fraction. Tb. Th, Trabecular thickness; Tb. Sp, Trabecular bone separation. (n=3; **P < 0.01, ***P < 0.001). The symbol ns means “no significance”.

To observe the effects of *P. gingivalis* OMVs in different growth stages *in vivo*, we constructed a periodontitis model in rats with ligated wires. After acclimatization for one week, 36 male rats were randomly divided into 6 groups, namely unligation group, PBS +ligation group, pre-log OMVs + ligation group, late-log OMVs +ligation group, stationary OMVs +ligation group, and *P. gingivalis* + ligation group (**Figure 8A**). After one month of treatment, it was discovered that, when compared to PBS + ligation group, rats with local injection of *P. gingivalis* OMVs and *P. gingivalis* (W83) had considerably more extensive alveolar bone abnormalities (**Figure 8B**). The height of bone resorption was almost double that of the PBS + ligation group (**Figures 8B–D**). Analysis of the results of Micro CT showed that the periodontal damage of pre-log OMVs had no significant difference compared with the PBS + ligation group, while the late-log and stationary OMVs groups showed almost the same degree of alveolar bone loss as the positive control group (*P. gingivalis* +ligation group) (**Figures 8B–D**). The cement enamel junction-alveolar bone crest (CEJ-ABC) distance was wider in the late-log and stationary OMVs groups than in the simple ligation and pre-log OMVs groups (**Figure 8D**). Meanwhile, a substantially decreased bone volume relative to total tissue volume (BV/TV) (**Figure 8C**) and trabecular thickness (Tb. Th) (**Figure 8E**) around the first molar were detected, while trabecular separation (Tb. Sp) were not profoundly impacted in this study (**Figure 8F**). The alveolar bone structure deterioration and osteoporosis were more severe in the late-log OMVs group, stationary OMVs group, and *P. gingivalis* group than those of the pre-log OMVs group, according to hematoxylin-eosin staining (HE staining) (**Figure 8G**). Additionally, the results of tissue section tartrate-resistant acid phosphatase (TRAP staining) revealed that the late-log OMVs group and *P. gingivalis* group had considerably more osteoclasts than the control group (**Figure 8H**). Nevertheless, it was worth noting that the stationary OMVs group had essentially no positive osteoclasts, which was contradictory with the degree of periodontal damage. We assumed that the severe loss of bone tissue interferes with the attachment of osteoclast, leading to abnormal bone metabolism.

In general, the results of the *in vivo* experiments showed the same trend as those of the *in vitro* experiments. The *P. gingivalis* OMVs groups had more serious damage to the periodontium than that of rats in the PBS group. However, further research is required to elucidate whether the main impairment was induced by the late-log and stationary OMVs or the interaction among all stages.

## 4 Discussion

As research has progressed in recent years, with the deepening of research, the OMVs gradually get into people’s sight as a virulent component of bacteria. On the one hand, their interactions with the body have become diverse and sophisticated owing to their complex components, and architectures; On the other hand, OMVs, as natural, highly immunogenic spherical nanoparticles, have potential value in the fields of targeted drugs and vaccines (Macdonald and Kuehn, 2013; Bitto and Kaparakis-Liaskos, 2017). We identified that *P. gingivalis* OMVs in the late-log and stationary stages were more toxic to host cells and can elicit stronger host immune responses than pre-log OMVs by analyzing their effects on macrophages, PDLSCs, and a periodontitis model. As a result, we believe that, in addition to focusing on the manipulation of physicochemical and even biological properties of OMVs via culture conditions and genetic engineering, the differences among OMVs produced in the different stages may bring some new insights to this field.

In this study, the size of the *P. gingivalis* OMVs isolated in different growth stages were found to be homogeneous, with no evident contaminants. The morphology of the pre-log OMVs was more variable than the other two categories of OMVs, with oval and elongated shapes (**Figure 1D**). As we expected that both BCA assay and NTA revealed that as culture time increased, the amount of OMVs rose, which was consistent with the previous studies, like *H. pylori*, *Pseudomonas aeruginosa*, and *E. coli* (Tashiro et al., 2010; McCaig et al., 2013; Zavan et al., 2019; Sharif et al., 2021).

When the *P. gingivalis* OMVs of each group were detected by SDS-PAGE, the proteins were different in the bands of 15-35 KDa and 55-75 KDa. The iTRAQ results confirmed this result, with GO annotations showing that late-log and stationary exhibited more upregulation in metabolic processes, cellular processes, cellular anatomical entities, catalytic activity, and binding proteins than pre-log OMVs protein (**Figure 2B**). In the KEGG metabolic pathway enrichment analysis, starch and sucrose metabolism-related proteins were more enriched in pre-log OMVs than the other two groups (**Figure 2C**), which may be related to the vigorous bacterial metabolism in this growth stage. Currently, OMVs have been observed to facilitate horizontal gene transfer, such as resistance gene transfer (Rumbo et al., 2011; Cooke et al., 2019; Aktar et al., 2021). The OMVs of *Acinetobacter baumannii* can export genes with resistance to penicillin and cephalosporins from the original bacteria to other bacteria (Rumbo et al., 2011; Liao et al., 2015). The presence of penicillin-resistance genes can be detected in the OMVs of *Neisseria gonorrhoeae*, suggesting that OMVs may transfer resistance genes to antibiotic-sensitive bacteria (Leduc et al., 2020). One of the more significant findings to emerge from this study is that we found higher drug resistance-related proteins in stationary OMVs, like cationic antimicrobial peptide resistance, β-lactam resistance, and vancomycin resistance. Therefore, it is necessary for us to further explore whether the OMVs in the stationary stage will play a greater role in horizontal gene transfer.

We then treated PDLSCs and macrophages with *P. gingivalis* OMVs in different growth stages to investigate the impact of OMVs on pathogenicity and host immune response. After 1 hour of treatment with PDLSCs, a minimal amount of adhesion and invasion was observed between OMVs and PDLSCs. Yet, in the treatment of macrophages, the majority of OMVs were internalized (**Figures 3A, B**). After 4 hours, a substantial percentage of OMVs were internalized both in PDLSCs and macrophages (**Figures 3C, D**). The research of *Fusobacterium nucleatum* OMVs yielded similar results. *Fusobacterium nucleatum* OMVs can enter macrophages in large numbers but seldom enter gingival fibroblasts (Chen et al., 2022).

In addition, macrophages were more sensitive to stimulation by *P. gingivalis* OMVs (**Figure 4A**) than PDLSCs, with 10 times higher inflammatory cytokines-related genes compared to PDLSCs (**Figure 7H**). When comparing *P. gingivalis* OMVs produced in different growth stages, we found that the stimulatory responses of cells to pre-log OMVs were not significantly different from those of the control group, while late-log and stationary OMVs significantly promoted PDLSCs and macrophages to express inflammatory cytokines-related genes. Previous studies have stimulated macrophages with LPS or live bacteria and found that the metabolic pattern of macrophages shifted to glycolysis, accompanied by a decrease in mitochondrial function and the reduction of the tricarboxylic acid cycle (TCA)-related gene expression (Garaude et al., 2016; Gleeson et al., 2016). Fleetwood et al. observed that *P. gingivalis* OMVs reduced the expression of TCA genes and glycolytic-related genes in macrophages (Fleetwood et al., 2017). However, when M1 macrophages are formed, their metabolism is mostly dependent on aerobic glycolysis, whereas M2 macrophages are the opposite. Therefore, *P. gingivalis* OMVs can thereby regulate inflammation by reprogramming glucose metabolism. Flow cytometry revealed that the proportion of M1-like macrophages in late-log and stationary OMVs groups exhibited 10 times higher than that in pre-log OMVs group (**Figure 4C**). The result of RT-qPCR also found evidence. The expression of M1 macrophages biomarkers-related genes was dramatically elevated in the late-log and stationary OMVs groups, yet marginally decreased in the pre-log OMVs group. M2 macrophage-related genes were not significantly altered (**Figure 4B**). In previous iTRAQ data, late-log OMVs had more proteins related to glucose metabolism than the other two groups, which may explain some of its regulation in macrophage polarization (**Figure 2C**).

PDLSCs proliferation, migration, and osteogenic activities were decreased to diverse degrees after stimulation by *P. gingivalis* OMVs produced in different growth stages, particularly in the stationary OMVs group (**Figure 5**). Additionally, different growth stages of *P. gingivalis* OMVs activated the NLRP3 inflammasomes (**Figure 7**), pro-caspase-1, and pro-IL-1β in PDLSCs, which are critical elements in periodontal tissue destruction. Elevated expression of pro-IL-1β up-regulated the expression of collagenolytic enzymes and matrix metalloproteinases in periodontal tissue, accompanied by the expression of receptor activator of NF-κB ligand (RANKL), which promoted the formation of osteoclasts, resulting in attachment loss (Zhang et al., 2020; Okamura et al., 2021). Late-log and stationary OMVs can initiate the formation of an inflammatory microenvironment in surrounding tissues by acting on both PDLSCs and macrophages. Simultaneously, the synergistic interaction of macrophages exacerbates the degree of PDLSCs damage.

Our *in vivo* experimental setting also allowed us to investigate the effect of late-log and stationary OMVs on the alveolar bone. *P. gingivalis* OMVs groups as expected induced alveolar bone loss, while the late-log and stationary OMVs groups had more obvious deterioration in alveolar bone micro-architecture compared with the pre-log OMVs groups, as proven by a markedly decreased BV/TV (**Figure 8C**) and Tb. Th (**Figure 8E**) around the first molar, in accordance with earlier relevant studies (Chen et al., 2022). Besides, CEJ-ABC (**Figure 8D**) value rose significantly in late-log and stationary OMVs groups. Tb. Sp (**Figure 8F**) was not significantly affected in this study. Interestingly, in both *in vivo* and *in vitro* experiments, pre-log OMVs generally exhibited low toxicity and even promoted proliferation and migration. A similar situation has occurred in previous studies. According to Xu, osteoblast autophagy can be triggered by low doses of LPS via activating the NF-B signaling pathway, which increased cell survival and proliferation while reducing apoptosis (Xu et al., 2018). Additionally, it has been proved that *P. gingivalis* OMVs can mediate LPS tolerance in monocyte/macrophage cell lines, regulate pro-inflammatory responses, and suppress the release of TNF-α, which may help *P. gingivalis* and other periodontopathogens elude host immune system (Duncan et al., 2004; Waller et al., 2016). However, the specific mechanism of pre-log OMVs promoting cell proliferation and migration requires further investigation.

In conclusion, this study showed that Sprague Dawley rats administered with late-log and stationary OMVs can induce more severe damage to the alveolar bone of rats. By boosting the formation of M1 macrophages and promoting the expression of inflammatory cytokines in macrophages, late-log and stationary OMVs contributed to the immunological response of the host. Simultaneously, they stimulate the NLRP3/IL-1β-related pathway of PDLSCs, suppressing cell proliferation, migration, and osteogenic differentiation, which prevents periodontal lesions from healing. This research significantly improves our understanding of the heterogeneity of natural OMVs in different growth stages. From a therapeutic point of view, periodontal inflammation can be reduced by specifically inhibiting *P. gingivalis* or its OMVs in the late-log growth stage; For drug research, the results of this study are more like the instruction manual of OMVs, which provides some reference for researchers when to isolate suitable OMVs. In brief, these outcomes could have a positive translational impact on the rational design of general microbiota-based therapeutics.

## 5 Limitations

Inflammatory response of host cells *in vivo* involves the participation of multiple microbial products in various signal pathways. In this study, we just conducted a preliminary exploration on the toxicity of OMVs to host, so further verification on the mechanism is needed. *In vivo* investigations with gene knockout rats, for example, may help us better comprehend the pathogenicity of *P. gingivalis* OMVs to the host. In addition, due to limitations in cultivation conditions, when collecting *P. gingivalis* OMVs of at different growth stages, the latter stage of vesicles includes the earlier stage-formed OMVs, which may have a certain impact on the discussion of quantitative results of outer membrane vesicles at different growth stages.

## Data availability statement

The original contributions presented in the study are included in the article/**Supplementary Material**, further inquiries can be directed to the corresponding author/s.

## Ethics statement

The studies involving humans were approved by the Ethics Committee of the affiliated Stomatological Hospital of Chongqing Medical University. The studies were conducted in accordance with the local legislation and institutional requirements. Written informed consent for participation in this study was provided by the participants’ legal guardians/next of kin. The animal studies were approved by the Experimental Animal Ethics Committee of Chongqing Medical University. The studies were conducted in accordance with the local legislation and institutional requirements. Written informed consent was obtained from the owners for the participation of their animals in this study.

## Author contributions

DY and HM designed the main study. HM and TG wrote the manuscript text. HM, TG, XQ, YS, SY conducted the research. HM, TG, XQ, YS, SY analyzed the data. DY critically revised the manuscript. All authors contributed to the article and approved the submitted version.
