# Supplementary material for: Bacterial growth stage determines the yields, protein composition, and periodontal pathogenicity of Porphyromonas gingivalis outer membrane vesicles
Source: Front Cell Infect Microbiol. 2023 Oct 11;13:1193198. doi: 10.3389/fcimb.2023.1193198 (PMC10602934; doi:10.3389/fcimb.2023.1193198)
Supplement: Supplementary file 1 [file Table_1.docx]

Supplementary Material

Bacterial growth stage determines the yields, protein composition, and periodontal pathogenicity of *Porphyromonas gingivalis* outer membrane vesicles

**Hongchen Mao1,2,3†, Ting Gong1,2,3†, Yuting Sun1,2,3, Shiyao Yang1,3,4, Xin Qiao1,2,3, Deqin Yang1,2,3***

**†: co-first author**: The author contributed equally to this work.

***: corresponding author**

*** Correspondence:**

**Dr. Deqin Yang**

**email:** [**yangdeqin@hospital.cqmu.edu.cn**](mailto:yangdeqin@hospital.cqmu.edu.cn)

# Table 1

**Table 1 Number of significant differences between pairwise samples**

| **Compared name** | **Total quant** | **Up-regulated** | **Total diff** | **Down-regulated** |
| --- | --- | --- | --- | --- |
| Pre-Log *P. gingivali*s OMVs vs Pre-Log *P. gingivalis* | 1183 | 326 | 628 | 302 |
| Late-Log *P. gingivali*s OMVs vs Late-Log *P. gingivalis* | 1183 | 339 | 622 | 283 |
| Stationary *P. gingivali*s OMVs vs Stationary *P. gingivalis* | 1183 | 370 | 710 | 340 |
| Pre-Log *P. gingivali*s OMVs vs Late-Log *P. gingivalis* OMVs | 1183 | 29 | 67 | 38 |
| Pre-Log *P. gingivali*s OMVs vs Stationary *P. gingivalis* OMVs | 1183 | 47 | 120 | 73 |
| Late-Log *P. gingivali*s OMVs vs Stationary *P. gingivalis* OMVs | 1183 | 52 | 139 | 87 |

# Supplementary Figures


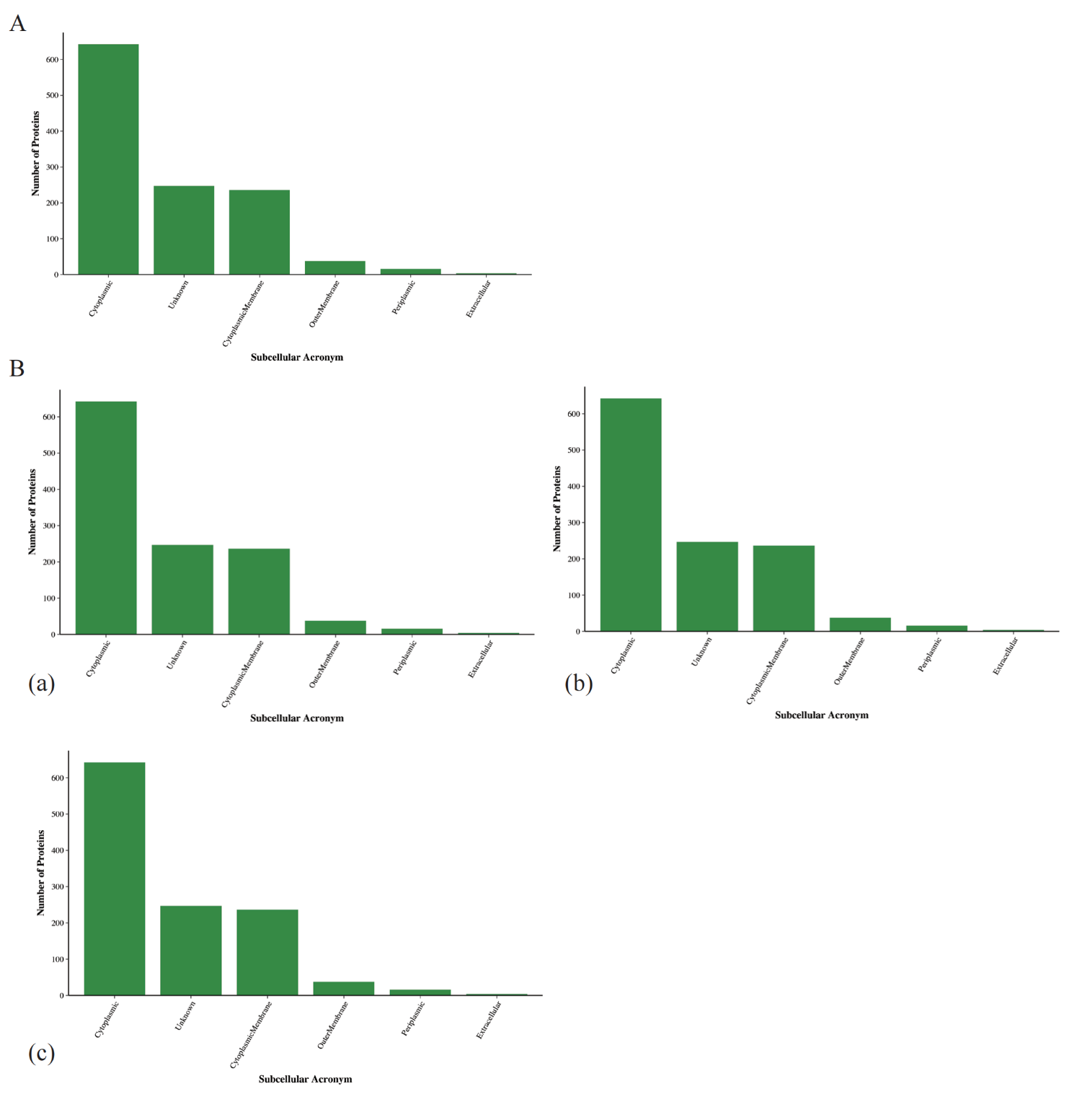


**Supplementary Figure 1.** Subcellular acronym of outer membrane vesicle proteins. (A) Results of the subcellular acronym of all identified proteins (X axis: the subcellular acronym, Y axis: the number of proteins); (B) Predictive analysis of subcellular acronym with differential proteins of OMVs in different growth stages. (a) Pre-Log OMVs vs Late-Log OMVs, (b) Pre-Log OMVs vs Stationary OMVs, (c) Late-Log OMVs vs Stationary OMVs.


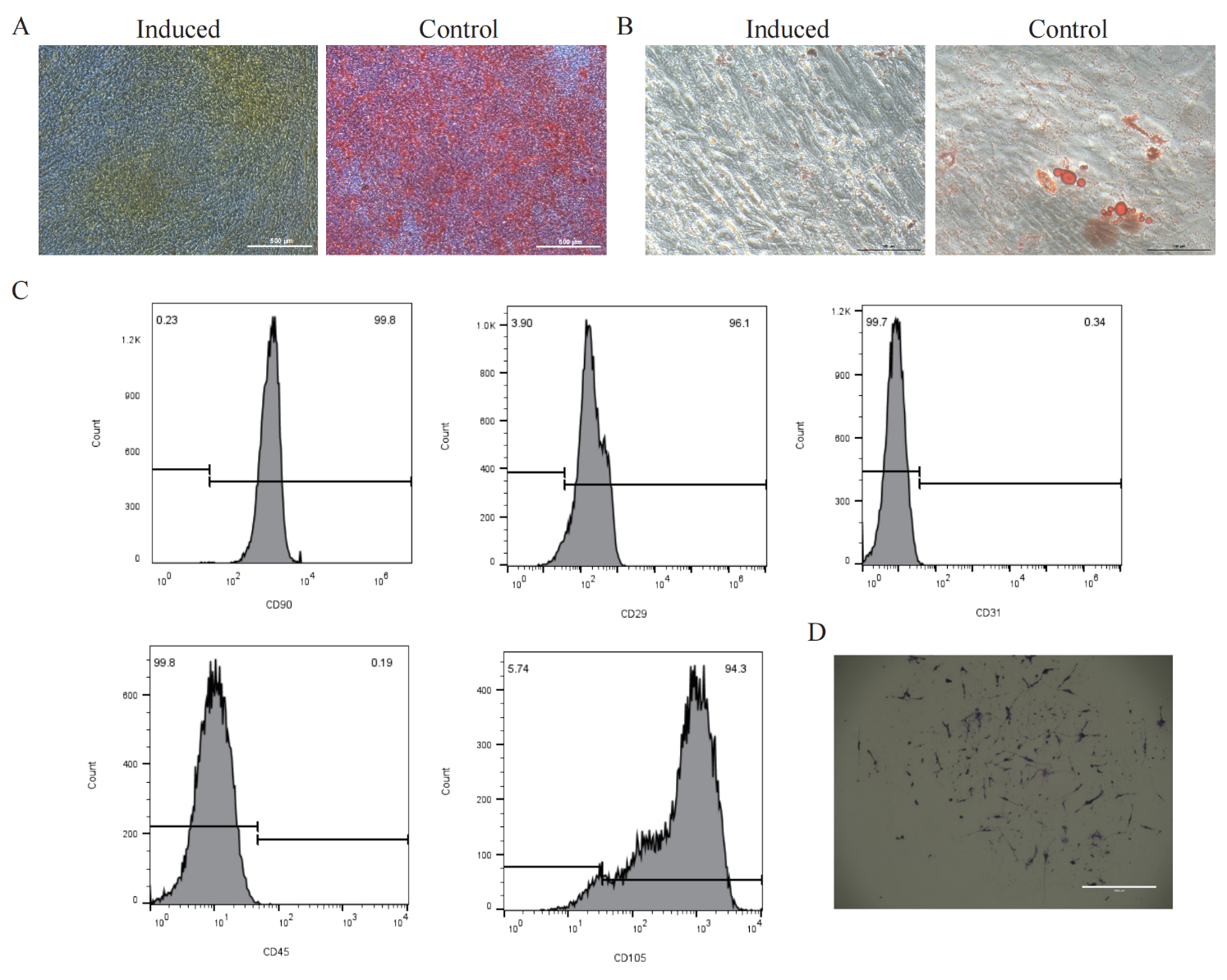


**Supplementary Figure 2.** Characterization of PDLSCs. **(A)** Alizarin red(ARS) staining of cellular matrix mineralization in PDLSCs; (Scale bar = 500 nm) **(B)** Oil Red O (ORO) staining of lipid droplet after PDLSCs adipogenic induction; (Scale bar = 500 nm) **(C)** Flow cytometric analysis of mesenchymal Stem Cells (MSCs) surface markers CD90, CD105, CD29, CD45, CD31 in PDLSCs. CD105 and CD90 are highly expressed, while CD29, CD45 and CD31 are low expressed. **(D)** Clonogenic assay to measure the proliferative ability of PDLSCs.
